# Economic burden of maternal depression among women with a low income in Cape Town, South Africa

**DOI:** 10.1192/bjo.2020.15

**Published:** 2020-04-03

**Authors:** Susan Cleary, Stacey Orangi, Emily Garman, Hanani Tabani, Marguerite Schneider, Crick Lund

**Affiliations:** University of Cape Town, Health Economics Unit, School of Public Health and Family Medicine, South Africa; KEMRI-Wellcome Trust Research Programme Nairobi, Health Economics Research Unit, Kenya; Alan J Flisher Centre for Public Mental Health, Department of Psychiatry and Mental Health, University of Cape Town, South Africa; School of Public Health, University of the Western Cape, South Africa; Alan J Flisher Centre for Public Mental Health, Department of Psychiatry and Mental Health, University of Cape Town, South Africa; Alan J Flisher Centre for Public Mental Health, Department of Psychiatry and Mental Health, University of Cape Town, South Africa

**Keywords:** Low and middle income countries, economic burden, maternal depression, societal costs

## Abstract

**Background:**

Maternal depression is a notable concern, yet little evidence exists on its economic burden in low- and middle-income countries.

**Aims:**

This study assessed societal costs and economic outcomes across pregnancy to 12 months postpartum comparing women with depression with those without depression. Trial registration: ClinicalTrials.gov: NCT01977326 (registered on 24 October 2013); Pan African Clinical Trials Registry (www.pactr.org): PACTR201403000676264 (registered on 11 October 2013).

**Method:**

Participants were recruited during the first antenatal visit to primary care clinics in Khayelitsha, Cape Town. In total, 2187 women were screened, and 419 women who were psychologically distressed were retained in the study. Women were interviewed at baseline, 8 months gestation and at 3 and 12 months postpartum; the Hamilton Rating Scale for Depression was used to categorise women as having depression or not having depression at each interview. Collected data included sociodemographics; health service costs; user fees; opportunity costs of accessing care; and travelling expenses for the women and their child(ren). Using Markov modelling, the incremental economic burden of maternal depression was estimated across the period.

**Results:**

At 12 months postpartum, women with depression were significantly more likely to be unemployed, to have lower per capita household income, to incur catastrophic costs and to be in a poorer socioeconomic group than those women without depression. Costs were higher for women with depression and their child(ren) at all time points. Modelled provider costs were US$805 among women without depression versus US$1303 in women with depression.

**Conclusions:**

Economic costs and outcomes were worse in perinatal women with depression. The development of interventions to reduce this burden is therefore of significant policy importance.

Maternal depression is a notable public health concern. In low- and middle-income countries (LMICs), the prevalence of maternal depression is estimated to be between 15 and 57%.^1^ In South Africa, the HIV epidemic, poverty, unplanned pregnancies, violence and a lack of support^2^ are argued to contribute towards prevalence rates of 16 and 39% in poor urban communities in Johannesburg^3^ and Cape Town,^3^ respectively, whereas rates as high as 47% have been reported in rural KwaZulu-Natal.^4^ In addition to the impact on the mother, maternal depression is associated with poor obstetric outcomes, including shorter gestation periods, reduced fetal neurodevelopment and low birth weight. Postnatally, maternal depression can compromise a mother's ability to care for her child with a potentially negative impact on cognitive, social, emotional and behavioural development.^5,6^

A strong association exists between depression and poverty,^7^ with recent evidence suggesting that two causal pathways are likely to act together to maintain this relationship.^8^ Under the social causation pathway, the conditions associated with poverty increase the risk for mental illness; whereas under the social selection or social drift pathway, those with mental illness are at increased risk of drifting into or remaining in poverty as a result of increased healthcare expenditure, reduced productivity and job loss.^7,9^

In South Africa, approximately 84% of the population uses healthcare services within the public health system. With the exception of in-patient care for higher income groups, public health services are free at the point of use and are financed via general taxation. A recent national survey estimated that 5% of these funds were spent on mental health services in total, of which 86% was spent within hospitals during the 2016/17 period.^10^ The analysis suggested a large potential treatment gap, with less than 7% of those in need using some form of out-patient mental health service during the period of analysis. Although the national survey added considerably to the knowledge base regarding expenditure on mental health related services in South Africa, it was unable to capture the utilisation of health services and the costs of care in those with maternal depression, given the lack of routinely collected diagnostic data for this group.

In addition to the key unknowns regarding expenditure on mental health related services for those with maternal depression, it is also unclear whether maternal depression has an impact on utilisation and expenditure for non-mental health related services. Following Prince et al,^11^ there are a number of hypothesised pathways through which depression may have an impact on the utilisation of health services. First, depression can lead to adverse health outcomes because of the increased risk factors for diseases that are associated with the biological effects of depression. Second, the persistent worry associated with depression may lead to overutilisation of healthcare. Third, depression may contribute towards poorer adherence to medication for existing physical conditions. And finally, worse physical health may lead to an increased risk of depression (for example learning one's HIV status can lead to feelings of despair and hopelessness).

Given the above gaps in the literature, the aim of this study is to model the economic burden of maternal depression from the time of first antenatal visit during pregnancy, until 12 months postpartum. In order to tease out the impact of maternal depression on costs and economic outcomes, we compare pregnant and postpartum women reporting no depression symptoms to those reporting symptoms of depression using a cut-off of 8 on the Hamilton Rating Scale for Depression (HRSD)^12^ and further estimate (a) the provider costs of services used by the women by depression status; (b) patient costs of accessing care, opportunity costs and out-of-pocket expenditure by depression status; and (c) the economic outcomes of the women by depression status, including comparisons of employment status, asset index based wealth, per capita household income and levels of catastrophic expenditure. Costs are reported for the women, and for their child(ren).

## Method

### Study design

This paper assesses the economic burden of maternal depression from a societal perspective^13^ by comparing patient costs, public provider costs and economic outcomes of pregnant and postpartum women according to their depression symptoms. Women were categorised as having depression if they scored >8 on the HRSD.^12^ Costs and economic outcomes were modelled over pregnancy and up to 12 months postpartum. Data for this analysis were collected within an individual-level randomised controlled trial. Trial registration: ClinicalTrials.gov: NCT01977326, registered on 24 October 2013; Pan African Clinical Trials Registry (www.pactr.org): PACTR201403000676264, registered on 11 October 2013.

The trial aimed to test a task-sharing intervention for women with symptoms of maternal depression. Women were randomised to one of two arms: (a) a structured manual-based psychological intervention including six counselling sessions conducted by community health workers trained in counselling techniques; or (b) enhanced usual care consisting of regular monthly telephone calls over a 3-month period, conducted by community health workers trained in conducting structured phone calls but not trained in counselling. Further details of the trial are available in published sources.^1^ Results from the trial indicted that the intervention had a non-significant impact on depression as measured by the HRSD in comparison with the control.^14^ For the purposes of this paper, which presents an analysis of the economic burden of maternal depression, all women in the trial were included, irrespective of arm.

Participants in this study were recruited at the Site B and Michael Mapongwana Midwife Obstetric Units (MOUs) in Khayelitsha, a peri-urban settlement on the outskirts of Cape Town. Many in Khayelitsha live in shacks without potable water, indoor sewage systems or electricity; the area is characterised by high rates of unemployment, crime and violence.^15^ Additional details of the study protocol are available in published sources.^1^ Recruitment was undertaken by trained field workers in collaboration with nurses providing antenatal services. Potential participants were pregnant women, aged 18 or older, at their first antenatal visit – typically in the second trimester of gestation.

Women who gave informed consent were recruited using a three-stage screening process: (a) verbal confirmation of the eligibility criteria for the study; (b) completion of a set of demographic questions; and (c) completion of the Edinburgh Postnatal Depression Scale (EPDS).

Women who scored 13 and above on the EPDS were recruited into the study, and were asked to complete a full baseline interview with a field worker who entered the responses into a mobile device linked to Mobenzi Researcher (a system that facilitates electronic data collection). If responses in this interview revealed signs of suicide ideation (scoring 17 or higher on the Mini International Neuropsychiatric Interview 6.0.0^16^), the woman was referred to the nursing sisters at the clinics for further assessment and possible onward referral to Khayelitsha District Hospital. In total, 2192 women were screened, of whom 425 screened positive on the EPDS and were enrolled. Of these, 6 were excluded when it was found that they did not meet inclusion criteria, and 419 remained in the study. In addition to the baseline assessment at first antenatal visit, women were interviewed again at 1 month before their due date, and at 3 and 12 months postpartum (four interviews in total).

Across these interviews, we collected a range of demographic, socioeconomic, health service utilisation and patient cost data, as further described below.

### Demographic, socioeconomic and health service utilisation data

In addition to baseline information such as median gestation, number of pregnancies, number of live births and educational status, at each interview we collected data on HRSD, employment status, partnership status, monthly household income and ownership or access to a range of assets and services.

In order to estimate the utilisation of health services, during each of the four interviews, women were asked about their utilisation of public ambulatory services in the preceding 3 months (including clinic and hospital out-patient department visits), and, if admitted to hospital, the number of in-patient days in the preceding 6 months. In addition, at their second interview (1 month before their due date), women were asked to estimate their utilisation of antenatal visits over their pregnancy; at their third interview (3 months postpartum) women reported the number of days spent at a health facility during labour and birth as well as their place of birth. In addition, women were asked about their utilisation of ambulatory and in-patient care for their child(ren) across the four interviews; at 12 months postpartum, women estimated the number of well-baby visits during the preceding year.

In order to estimate the opportunity cost of using services and out-of-pocket payments (i.e. the patient perspective), women were asked about travel, waiting and consultation times and about travelling expenses for each of their ambulatory visits in the preceding 3 months, for antenatal services over their pregnancy and for well-baby services in the first year after birth. As before, data were collected for services utilised by the women and by her child(ren). In the South African public health system, ambulatory services are free at the point of use, and fees for in-patient care differ depending on economic status. On the other hand, user fees are incurred for most private health services (for example private general practitioners). For the patient perspective, women were therefore asked about any user fees they had incurred across the range of public and private healthcare services utilised.

### Valuation of economic outcomes and costs

Household income was collected as a categorical variable (given that such a strategy has been shown to improve response rates^17^) and transformed into a quantitative variable using a regression approach, where household income was predicted as a function of baseline employment, education and socioeconomic status. Per capita household income was computed as total household income divided by the total number of household members. Following the approach of similar studies,^17,18^ socioeconomic status was estimated at each time point using multiple correspondence analysis across a range of variables relating to access to services, main source of income, whether income is fixed or variable, employment and education status, ownership and type of dwelling, type of shop where food is bought and access to banking services.

All costs were expressed in 2014/15 prices and converted to US dollars (US$1 = 11.69 South African Rand) using an average exchange rate over the same period.^19^ Provider and patient average unit costs were calculated using a number of approaches. For public health services, a gross costing approach was used to compute an average cost per clinic visit, per hospital out-patient department visit, per in-patient day, per delivery day at an MOU and per delivery day at a hospital, using audited expenditure and service utilisation data from all health facilities across the Western Cape Province.^20^ For clinics and community health centres, the overall expenditure was divided by the patient headcount over the same period; for hospitals, total expenditure was split into in-patient and out-patient unit costs using the patient day equivalent method, where the cost of an out-patient department visit is assumed to be one-third of the cost of an in-patient day.^10^ Once these average unit costs were computed, the costs of public health services for the women and their child(ren) were modelled by multiplying estimates of service utilisation (from the four interviews) against these unit costs. This modelling approach is described in more detail below.

As mentioned above, patient costs included travel, waiting and consultation times for the ambulatory services utilised by the women and their child(ren). Following the literature, the opportunity cost of time can be valued using wages/salary earnings foregone^21^ and is therefore based on assumed lost working hours. In this study, in order to value these costs equitably, the mean per capita income reported by the women at the baseline interview was used as a proxy of this opportunity cost. In other words, the opportunity cost of the time women spent accessing services for themselves and for their child(ren) was valued at the same rate. This opportunity cost was based on an assumed 228 working days per annum and 8 working hours per day. We did not include an opportunity cost of children's time (given that they do not work). In addition, we did not include an opportunity cost of time for delivery or in-patient care given that these times would likely include non-working hours. All other patient costs (travelling costs and user fees) were based on the values reported by respondents. In the analysis, estimates of time, travel and user fee costs were compared with the mean per capita income of the respondent's own household in order to assess the share of per capita household income spent on these costs.

### Modelling approach

**Fig. 1:**
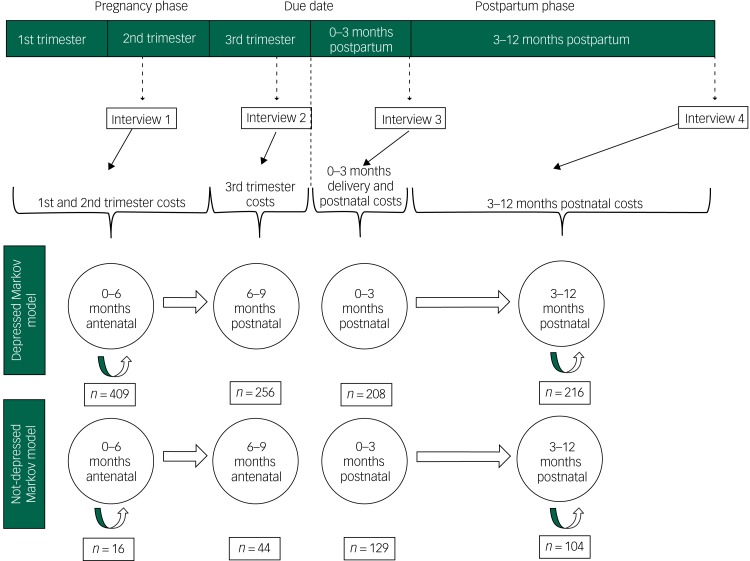
Relationship between timing of interview and assumptions made to model costs during pregnancy and up to 12 months postpartum.

Using Markov modelling with a 3-month cycle length, we have estimated the economic burden of maternal depression over pregnancy and up to 12 months postpartum by comparing women with depression symptoms to those without. In other words, responses from women at each of the four interviews were used to populate either a ‘depressed Markov model state’ or ‘not depressed Markov model state’ using a cut-off of 8 on the HRSD.^12^ Figure 1 illustrates the Markov modelling approach. For the purposes of this model, Markov states are developed to capture variability in costs and economic burden as reported by the women across the four interviews. As is shown, modelled participants enter the model at 0–6 months antenatal, remaining in this Markov state for two cycle lengths (i.e. 6 months). Thereafter, they move to 6–9 months antenatal, and 0–3 months postnatal, spending one cycle in each of these Markov states. Finally, they move to 3–12 months postnatal, and spend three cycles in that state. This is therefore not a longitudinal analysis of economic burden based on participants’ baseline depressive symptoms; such an approach was precluded by the low numbers of women without depression at baseline (*n* = 16, see Fig. 1). Instead, building on a Markov modelling approach, women are allocated to a ‘depressed Markov state’ or ‘non-depressed Markov state’ depending on their HRSD score at each interview in order to generate a modelled estimate of the patient and provider costs of women with and without depression and their child(ren) across pregnancy and up to 12 months postpartum. To do so, we have assumed that the estimates of service utilisation and patient costs reported in the baseline interview are applicable to the first and second trimesters of pregnancy; services and costs reported at 1 month before the due date are assumed to occur across the third trimester; services and costs reported at 3 months postnatal are assumed to apply to that period, including the costs of delivery; while services and costs reported at 12 months postnatal apply to the 3–12 months postnatal period.

As mentioned above, the utilisation of antenatal and well-baby care and the associated time, travel and user fee costs were collected at 1 month before the due date and at 12 months postpartum, respectively. The antenatal costs were spread across the pregnancy whereas the well-baby visit costs were spread across the postpartum period. This approach to utilising the interview data to estimate costs by depression status over the full period (pregnancy and 12 months postpartum) is also illustrated in Fig. 1.

For both the public provider and patient perspective, estimates of service utilisation and patient time, travel and user fee costs and opportunity costs were generated within StataSE 16, and then were entered into a Markov model created in Treeage Pro 2013. Separate models were created for estimating the costs for mothers and their child(ren) within both the provider and patient perspectives and for participants with and without depression, based on their HRSD scores at each time point. In order to assess data variability, the estimated means and s.d. were entered as gamma distributions in the models. Each model was run 10 000 times in order to capture data uncertainty using probabilistic sensitivity analysis. Where *P*-values are presented, these have been derived using the Kruskal–Wallis comparison of means test for quantitative data and the Pearson's χ^2^-test for categorical data.

### Ethical approval

The study obtained ethical approval from the University of Cape Town Human Research Ethics Committee (Ref no 226/2011 and 469/2015) and the National Institute of Mental Health Data Safety and Monitoring Board. All participants provided written informed consent.

## Results

### Participant characteristics

Table 1 presents clinical, demographic and socioeconomic characteristics of the participants at baseline and, where relevant, across each interview. Data are compared between the participants with and without depression, based on responses to the HRSD at each interview. As shown, there were no significant differences in age, duration of gestation, number of pregnancies or number of live births between the two groups at baseline. There were also no significant differences in education status. Over time, there were significant differences in employment, household income and socioeconomic status. At 1 month before the due date, 56% (*n* = 256) of the depression group reported being unemployed versus 41% (*n* = 44) in the non-depression group (*P* = 0.01). Similarly, at 12 months postpartum, 51% (*n* = 216) of the depression group was unemployed in comparison to 35% (*n* = 104) in the non-depression group (*P* = 0.02). Mean per capita monthly household income was significantly higher in the non-depression group at 12 months postpartum (US$59.84 *v*. US$52.35; *P* = 0.03) and women without depression were more likely to be in the richer SES group (62% *v*. 50% for the non-depression group versus depression group; *P* = 0.04). Although not significantly different by depression status, there was a significant decrease in women reporting a partner across the study period, changing from 75% at baseline to 57% at 12 months postpartum (*P* < 0.001).

**Table 1:** Patient clinical, demographic and socioeconomic characteristics during the perinatal period by depression status

	1st ANC visit			1 month before due date			3 months postpartum			12 months postpartum		
	Non-depression group	Depression group	*P*	Non-depression group	Depression group	*P*	Non-depression group	Depression group	*P*	Non-depression group	Depression group	*P*
*n* (%)	16 (4)	409 (96)		44 (15)	256 (85)	–	129 (38)	208 (62)	–	104 (33)	216 (68)	–
Age, years: mean	28.5	27.0	0.12	–	–	–	–	–	–	–	–	–
Gestation at recruitment, weeks: median	16	18	0.11	–	–	–	–	–	–	–	–	–
Number of pregnancies, median	2	2	0.79	–	–	–	–	–	–	–	–	–
Number of live births, median	1	1	0.80	–	–	–	–	–	–	–	–	–
Highest education level completed, %												
Grade 0–11	56.25	59.17	0.97	–	–	–	–	–	–	–	–	–
Grade 12	37.50	35.45		–	–	–	–	–	–	–	–	–
Post-schooling	6.25	5.38		–	–	–	–	–	–	–	–	–
Self-reported HIV status, %												
Negative	60.00	68.08	0.69	65.91	67.72	0.81	64.34	67.79	0.52	61.54	66.20	0.41
Positive	40.00	30.67		34.09	32.28		35.66	32.21		38.46	33.80	
Refused	0.00	1.25		0.00	0.00		0.00	0.00		0.00	0.00	
Employment status, %												
Working for pay	31.25	35.21	0.29	34.09	33.59	**0.01**	28.68	26.44	0.55	45.19	33.33	**0.02**
Self-employed/piece jobs	18.75	10.02		15.91	4.30		4.65	5.77		11.54	11.11	
Unemployed	50.00	40.83		40.91	56.25		59.69	63.94		34.62	51.39	
Studying	0.00	13.94		9.09	5.86		6.98	3.85		8.65	4.17	
Partnership status, %												
Has partner, live together	37.50	34.72	0.95	31.82	36.72	0.33	30.23	35.10	0.54	28.85	29.17	0.97
Has partner, live apart	62.50	63.33		59.09	55.86		55.81	50.96		28.85	26.85	
No partner, lives with family	0.00	1.47		9.09	4.30		10.85	12.50		39.42	40.28	
No partner, lives alone	0.00-	0.49		0.00	3.12		3.10	1.44		2.88	3.70	
Monthly household income, mean per capita	77.07	76.73	0.61	86.93	69.49	0.18	58.40	52.61	0.07	59.84	52.35	**0.03**
In richer SES group, %	56.25	49.63	0.60	45.45	55.86	0.20	54.26	52.88	0.81	61.54	49.54	**0.04**

ANC, antenatal care services; SES, socioeconomic status.

### Public provider costs

Supplementary Table 1 (available at https://doi.org/10.1192/bjo.2020.15) presents the estimates of public health service utilisation for the women and their child(ren) across the four interviews. All data are presented per 3-month period. As mentioned, the costs of these services have been valued using a gross costing approach, applied to publicly available audited expenditure and service utilisation data from the Western Cape Government.^20^ Within the Markov model, estimates of service utilisation are multiplied against the relevant unit costs in order to calculate the mean cost over the pregnancy and up to 12 months postpartum for the non-depression group versus the depression group. The results of the probabilistic sensitivity analysis are captured as 90% uncertainty intervals around the mean results.

**Fig. 2:**
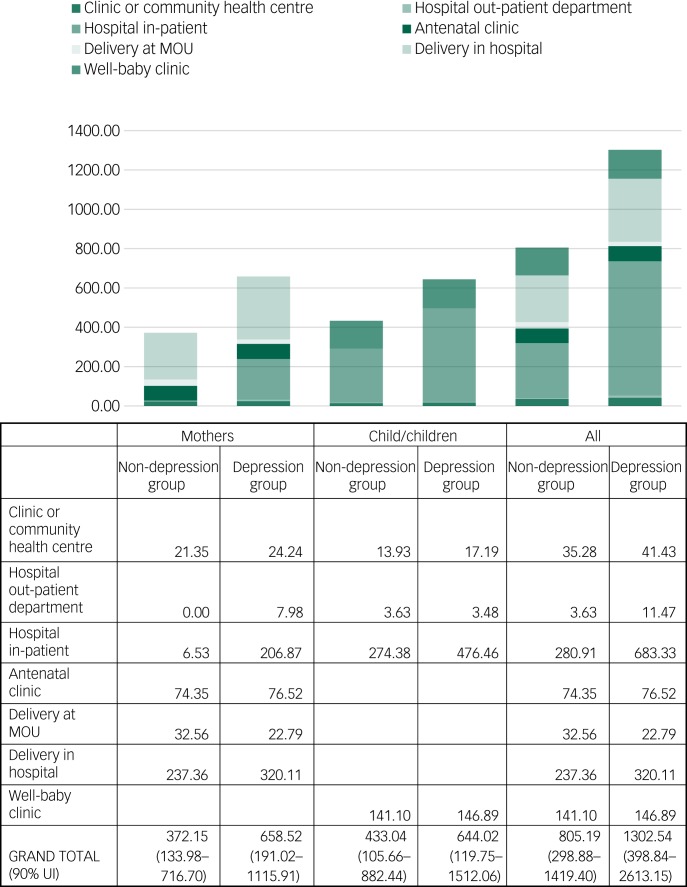
Public provider costs of care for mothers and their child(ren) (US$) by depression status.

MOU, Midwife Obstetric Unit; UI, uncertainty interval.

As shown in Fig. 2 and supplementary Table 2, the mean results indicate that public provider costs are higher for the mothers with depression, at US$659 in comparison with US$372; for children the mean cost is US$644 *v*. US$433 respectively. The main drivers of these differences are the utilisation of in-patient care (for the mother and for her child(ren)) and deliveries within a hospital instead of a MOU. On the other hand, similar costs are incurred for antenatal and well-baby services. These results should, however, be interpreted with caution given the wide variation in estimates, as captured within 90% uncertainty intervals using probabilistic sensitivity analysis.

### Patient costs

**Fig. 3:**
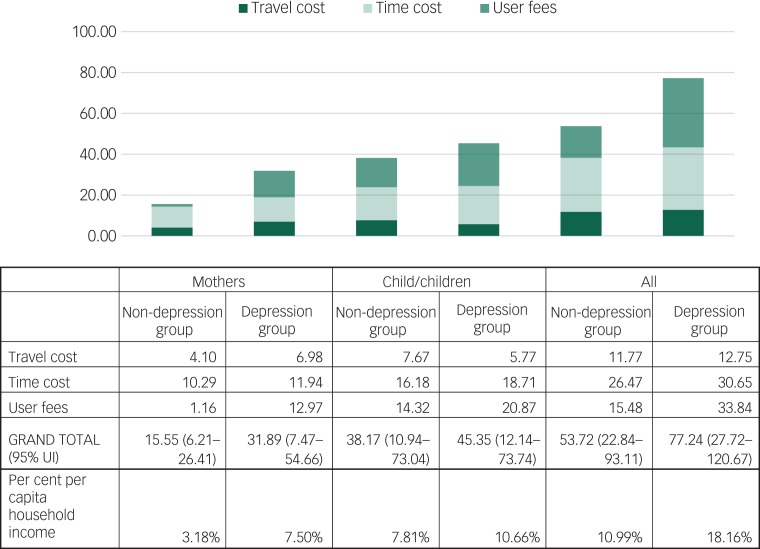
Patient costs of accessing care for mothers and their child(ren) by depression status (US$).

Supplementary Table 3 presents the travel and time costs, and user fee estimates that are used to derive the patient cost results that are presented in Fig. 3. The latter suggests a similar trend of higher costs in the depression group. Although patient costs are far lower than provider costs, these costs are nevertheless significant from a household perspective; in total, 11 and 18% of per capita household income is spent in accessing services for the women and their children in the non-depression group and depression group, respectively. However, as before, the overlapping uncertainty intervals suggest that – although costs are higher in the depression group – there may not be significant differences between the groups based on their depressive symptoms.

UI, uncertainty interval.

## Discussion

### Main findings

This study estimated the economic burden of maternal depression in participants who are psychologically distressed in a low-income setting. At 12 months postpartum, women without depression were significantly more likely to be employed, to have a higher household income and to fall into the wealthier socioeconomic group. In contrast, provider and patient costs were higher for the women and their child(ren) who were categorised as being in the depression group across all time points. Although patient costs are markedly lower than provider costs, catastrophic spending levels were evident, with 18% *v.* 11% of per capita household income spent accessing health services in the depression versus non-depression groups. Although the wide variations in these cost estimates generated overlapping uncertainty intervals, the consistent trend in higher costs for women with depression and their children is of significant policy relevance.

### Interpretation of our findings

Prince et al^11^ have suggested a number of pathways through which mental health may have an impact on physical health, and vice versa. The detailed utilisation data collected in this study may allow some insights to be generated about which of these pathways might operate in this setting. First, there was a correlation between depression status and utilisation of in-patient care, and this was the case for the mothers and their children. Second, there was a correlation between depression status and hospital-based deliveries (likely to be more complicated than MOU deliveries). Both of these correlations provide support to the notion that worse physical health may encourage depression and/or that depression might encourage worse physical health. In contrast there was no correlation between depression status and utilisation of antenatal and well-baby services, suggesting that it is not the case that the persistent worry associated with depression leads to an overutilisation of healthcare in this setting.

### Limitations

This study has a number of shortcomings. First, the study is a subanalysis of a group of women receiving a psychological intervention or enhanced usual care within a randomised controlled trial. Given that these women are trial participants, they are unlikely to be representative of all pregnant women accessing antenatal services in this setting, or other settings. In earlier analyses, we explored using the control arm (enhanced usual care) to derive the estimates for this paper and found similar patterns of higher costs in the depression group. Therefore, to maximise our sample size, we have included all women from the trial in this analysis. Despite this strategy, there are wide variations in our estimates that we have assessed via probabilistic sensitivity analysis and have presented as uncertainty intervals around mean costs.

Second, we have used a Markov modelling framework to compare costs between participants with depression versus participants without depression at each time point. In reality, it is likely that the depressive symptoms of women would change over time, which means that the total costs calculated for the depression versus non-depression group over the full time period should be interpreted cautiously. Although an alternative approach would have been to assess economic burden using baseline depression status, the small sample size of the non-depression group at baseline (*n* = 16), coupled with loss to follow-up over time, precluded the use of such an approach. Future research could improve on our estimates by including probabilities of becoming more or less depressed over the study period, thereby generating a more accurate estimate of total costs for this group of women as a whole. This would also facilitate an understanding of the cost-effectiveness of interventions to alleviate depression in pregnant and postpartum women.

Third, for public sector services from the provider perspective, we have used routine expenditure and patient data from the Western Cape Department of Health to calculate average unit costs. Although such an approach is crude, it is necessary given the absence of differentiated unit costs for different types of services (for example in-patient care in a surgical ward versus in-patient care in a general medical ward). Moreover, we have based our estimates of service utilisation on self-reports from participants. Without electronic patient data linked to a unique patient identifier, we are unable to follow patients up at clinics and hospitals to conduct nuanced primary costing for the type of service utilised. This would in addition be prohibitively time consuming and costly.

### Implications

In conclusion, to the best of our knowledge this study is the first to assess the economic burden of maternal depression across pregnancy and up to 12 months postpartum in a LMIC setting. Our results strongly suggest higher patient and provider costs in women who are more depressed. In addition, less favourable economic outcomes (employment, household income and socioeconomic status) were evident in women with depression at 12 months postpartum. While maternal depression is currently underdiagnosed and undertreated in South Africa, it is nevertheless associated with high costs. The potential to avert some of these costs through intervening to reduce this burden of maternal depression is therefore of significant policy importance.

## Data Availability

The authors have access to deidentified data in order to do the analysis. The data collected for this study is available from the corresponding author upon reasonable request. Deidentified participant data will be made publicly available from June 2020.

## Supplementary material

For supplementary material accompanying this paper visit http://dx.doi.org/10.1192/bjo.2020.15. click here to view supplementary material
