# Supplementary material for: Economic burden of maternal depression among women with a low income in Cape Town, South Africa
Source: BJPsych Open. 2020 Apr 3;6(3):e36. doi: 10.1192/bjo.2020.15 (PMC7176833; doi:10.1192/bjo.2020.15)
Supplement: Supplementary file 1 [file S2056472420000150sup001.docx]

**Supplementary Table 1: Estimates of public health service utilisation per three month period**

|  | **MOTHERS** | | | | **CHILD/CHILDREN** | | | |
| --- | --- | --- | --- | --- | --- | --- | --- | --- |
|  | **Not depressed** | | **Depressed** | | **Not depressed** | | **Depressed** | |
| **First ANC visit** | mean | SD | mean | SD | mean | SD | mean | SD |
| Clinic or community health centre visits | 0.31 | 0.70 | 0.43 | 0.78 | 0.00 | 0.00 | 0.07 | 0.27 |
| Hospital outpatient department visits | 0.00 | 0.00 | 0.02 | 0.20 | 0.00 | 0.00 | 0.00 | 0.05 |
| Hospital inpatient days | 0.00 | 0.00 | 0.12 | 0.77 | 0.00 | 0.00 | 0.14 | 1.57 |
| **1 month before due date** |  |  |  |  |  |  |  |  |
| Clinic or community health centre visits | 0.09 | 0.47 | 0.06 | 0.37 | 0.00 | 0.00 | 0.03 | 0.17 |
| Hospital outpatient department visits | 0.00 | 0.00 | 0.02 | 0.22 | 0.00 | 0.00 | 0.00 | 0.06 |
| Hospital inpatient days | 0.01 | 0.08 | 0.29 | 2.22 | 0.00 | 0.00 | 0.01 | 0.08 |
| Antenatal clinic visits | 1.37 | 0.50 | 1.41 | 0.56 | N/A |  |  |  |
| **3 months postpartum** |  |  |  |  |  |  |  |  |
| Clinic or community health centre visits | 0.08 | 0.55 | 0.06 | 0.23 | 0.02 | 0.12 | 0.12 | 0.41 |
| Hospital outpatient department visits | 0.00 | 0.00 | 0.02 | 0.22 | 0.02 | 0.12 | 0.01 | 0.10 |
| Hospital inpatient days | 0.02 | 0.22 | 0.36 | 3.46 | 0.21 | 0.83 | 0.49 | 2.73 |
| MOU delivery days | 0.60 | 0.73 | 0.42 | 0.70 | N/A |  |  |  |
| Hospital delivery days | 1.09 | 1.16 | 1.47 | 1.21 | N/A |  |  |  |
| **12 months postpartum** |  |  |  |  |  |  |  |  |
| Clinic or community health centre visits | 0.13 | 0.34 | 0.12 | 0.33 | 0.25 | 0.55 | 0.22 | 0.57 |
| Hospital outpatient department visits | 0.00 | 0.00 | 0.01 | 0.12 | 0.01 | 0.10 | 0.01 | 0.10 |
| Hospital inpatient days | 0.00 | 0.00 | 0.02 | 0.31 | 0.35 | 1.34 | 0.47 | 1.49 |
| Well baby visits | N/A |  |  |  | 1.95 | 0.67 | 2.03 | 0.66 |

Depression status was based on a cutoff of 8 on the HDRS, and was assessed at each timepoint.

**Supplementary Table 2: Public provider costs during pregnancy and up to 12 months postpartum (US$)***

|  | **MOTHERS** | | **CHILD/CHILDREN** | | **ALL** | |
| --- | --- | --- | --- | --- | --- | --- |
|  | **Not depressed** | **Depressed** | **Not depressed** | **Depressed** | **Not depressed** | **Depressed** |
| **First and second trimester** |  |  |  |  |  |  |
| Clinic or health centre | 11.22 | 15.56 | 0.00 | 2.53 | 11.22 | 18.09 |
| Hospital outpatient | 0.00 | 2.90 | 0.00 | 0.29 | 0.00 | 3.19 |
| Hospital inpatient | 0.00 | 52.26 | 0.00 | 60.97 | 0.00 | 113.24 |
| Antenatal clinic | 49.57 | 51.01 | N/A | N/A | 49.57 | 51.01 |
| Total | 60.78 | 121.74 | 0.00 | 63.80 | 60.78 | 185.53 |
| **Third trimester** |  |  |  |  |  |  |
| Clinic or health centre | 1.63 | 1.09 | 0.00 | 0.54 | 1.63 | 1.63 |
| Hospital outpatient | 0.00 | 1.45 | 0.00 | 0.29 | 0.00 | 1.74 |
| Hospital inpatient | 2.18 | 63.15 | 0.00 | 1.74 | 2.18 | 64.89 |
| Antenatal clinic | 24.78 | 25.51 |  |  | 24.78 | 25.51 |
| Total | 28.59 | 91.19 | 0.00 | 2.58 | 28.59 | 93.77 |
| **0-3 months postpartum** |  |  |  |  |  |  |
| Clinic or health centre | 1.45 | 1.09 | 0.36 | 2.17 | 1.81 | 3.26 |
| Hospital outpatient | 0.00 | 1.45 | 1.45 | 0.73 | 1.45 | 2.18 |
| Hospital inpatient | 4.36 | 78.39 | 45.73 | 106.70 | 50.08 | 185.10 |
| Delivery at MOU | 32.56 | 22.79 | N/A | N/A | 32.56 | 22.79 |
| Delivery in hospital | 237.36 | 320.11 | N/A | N/A | 237.36 | 320.11 |
| Well baby clinic |  |  | 35.28 | 36.72 | 35.28 | 36.72 |
| Total | 275.72 | 423.83 | 82.82 | 146.32 | 358.54 | 570.15 |
| **3-12 months postpartum** |  |  |  |  |  |  |
| Clinic or health centre | 7.06 | 6.51 | 13.57 | 11.94 | 20.62 | 18.45 |
| Hospital outpatient | 0.00 | 2.18 | 2.18 | 2.18 | 2.18 | 4.36 |
| Hospital inpatient | 0.00 | 13.07 | 228.65 | 307.04 | 228.65 | 320.11 |
| Well baby clinic | N/A | N/A | 105.83 | 110.17 | 105.83 | 110.17 |
| Total | 7.06 | 21.76 | 350.22 | 431.33 | 357.27 | 453.08 |
| **GRAND TOTAL (90% UI)** | **372,15 (133.98-716.70)** | **658,52 (191.02-1115.91)** | **433,04 (105.66-882.44)** | **644,02 (119.75-1512.06)** | **805,19 (298,88-1419,40)** | **1302,54 (398.84-2613.15)** |

*Costs are calculated from estimates of service utilisation multiplied by unit costs where: cost per clinic or community health centre visit = US$18.09; cost per hospital outpatient department visit = US$72.59; cost per hospital inpatient day = US$217.76; cost per MOU delivery day = US$54.27

Source: (22)

**Supplementary Table 3: Estimates of private health service utilisation, patient time, travel costs and user fee payments per 3-month period**

|  | **MOTHERS** | | | | **CHILD/CHILDREN** | | | | | | |
| --- | --- | --- | --- | --- | --- | --- | --- | --- | --- | --- | --- |
|  | **Not depressed** | | **Depressed** | | **Not depressed** | | | **Depressed** | | |  |
| **First ANC visit** | mean | SD | mean | SD | mean | SD | mean | | SD |  |  |
| GP visits (utilisation) | 0.06 | 0.25 | 0.11 | 0.34 | 0.00 | 0.00 | 0.12 | | 0.11 |  |  |
| Private IP days (utilisation) | 0.00 | 0.00 | 0.00 | 0.00 | 0.00 | 0.00 | 0.00 | | 0.00 |  |  |
| Travel, waiting and consultation times (minutes) | 47.00 | 92.80 | 89.37 | 167.01 | 0.00 | 0.00 | 9.74 | | 41.40 |  |  |
| Travel costs (US$) | 0.42 | 0.90 | 0.66 | 2.48 | 0.00 | 0.00 | 0.06 | | 0.33 |  |  |
| User fees (US$) | 0.00 | 0.00 | 2.03 | 8.62 | 0.00 | 0.00 | 3.23 | | 61.34 |  |  |
| **1 month before due date** |  |  |  |  |  |  |  | |  |  |  |
| GP visits (utilisation) | 0.02 | 0.15 | 0.05 | 0.22 | 0.00 | 0.00 | 0.01 | | 0.09 |  |  |
| Private IP days (utilisation) | 0.00 | 0.00 | 0.00 | 0.00 | 0.00 | 0.00 | 0.00 | | 0.00 |  |  |
| Travel, waiting and consultation times (minutes) | 19.66 | 96.39 | 23.92 | 85.23 | 0.00 | 0.00 | 4.69 | | 25.44 |  |  |
| ANC travel, waiting and consultation times (minutes) | 791.36 | 510.27 | 846.25 | 785.53 | N/A |  |  | |  |  |  |
| Travel costs (US$) | 0.07 | 0.34 | 0.17 | 0.87 | 0.00 | 0.00 | 0.05 | | 0.39 |  |  |
| ANC travel costs (US$) | 0.84 | 1.05 | 1.58 | 2.27 | N/A |  |  | |  |  |  |
| User fees (US$) | 0.87 | 5.80 | 1.98 | 15.38 | 0.00 | 0.00 | 0.26 | | 3.03 |  |  |
| ANC user fees (US$) | 0.00 | 0.00 | 1.65 | 21.08 | N/A |  |  | |  |  |  |
| **3 months postpartum** |  |  |  |  |  |  |  | |  |  |  |
| GP visits (utilisation) | 0.16 | 0.18 | 0.05 | 0.24 | 0.04 | 0.23 | 0.04 | | 0.19 |  |  |
| Private IP days (utilisation) | 0.00 | 0.00 | 0.01 | 0.07 | 0.00 | 0.00 | 0.00 | | 0.00 |  |  |
| Travel, waiting and consultation times (minutes) | 14.96 | 84.31 | 23.00 | 77.79 | 13.78 | 65.28 | 27.09 | | 86.67 |  |  |
| Travel costs (US$) | 0.13 | 0.83 | 0.21 | 0.84 | 0.08 | 0.42 | 0.15 | | 0.57 |  |  |
| User fees (US$) | 0.29 | 3.32 | 0.75 | 4.32 | 0.21 | 1.13 | 0.73 | | 3.55 |  |  |
| **12 months postpartum** |  |  |  |  |  |  |  | |  |  |  |
| GP visits (utilisation) | 0.00 | 0.00 | 0.02 | 0.14 | 0.24 | 0.68 | 0.18 | | 0.55 |  |  |
| Private IP days (utilisation) | 0.00 | 0.00 | 0.00 | 0.00 | 0.00 | 0.00 | 0.01 | | 0.10 |  |  |
| Travel, waiting and consultation times (minutes) | 35.99 | 101.00 | 40.61 | 107.34 | 55.93 | 106.22 | 53.25 | | 102.35 |  |  |
| Well baby travel, waiting and consultation times (minutes) | N/A |  |  |  | 1434.71 | 735.56 | 1660.02 | | 951.30 |  |  |
| Travel costs (US$) | 0.18 | 1.19 | 0.18 | 0.63 | 0.49 | 1.28 | 0.47 | | 1.20 |  |  |
| Well baby travel costs (US$) | N/A |  |  |  | 1.53 | 3.79 | 1.01 | | 1.98 |  |  |
| User fees (US$) | 0.00 | 0.00 | 0.41 | 2.68 | 3.97 | 11.76 | 2.30 | | 9.36 |  |  |
| Well baby user fees (US$) | N/A |  |  |  | 0.55 | 5.61 | 1.63 | | 9.67 |  |  |
